# Analysis of immune cell populations in atrial myocardium of patients with atrial fibrillation or sinus rhythm

**DOI:** 10.1371/journal.pone.0172691

**Published:** 2017-02-22

**Authors:** Natalia Smorodinova, Martin Bláha, Vojtěch Melenovský, Karolína Rozsívalová, Jaromír Přidal, Mária Ďurišová, Jan Pirk, Josef Kautzner, Tomáš Kučera

**Affiliations:** 1 Institute of Histology and Embryology, The First Faculty of Medicine, Charles University, Prague, Czech Republic; 2 Institute for Clinical and Experimental Medicine-IKEM, Department of Cardiology, Prague, Czech Republic; 3 Institute for Clinical and Experimental Medicine-IKEM, Department of Cardiovascular Surgery, Prague, Czech Republic; Centro Cardiologico Monzino, ITALY

## Abstract

**Background:**

Atrial fibrillation (AF) is the most common arrhythmia and despite obvious clinical importance remains its pathogenesis only partially explained. A relation between inflammation and AF has been suggested by findings of increased inflammatory markers in AF patients.

**Objective:**

The goal of this study was to characterize morphologically and functionally CD45-positive inflammatory cell populations in atrial myocardium of patients with AF as compared to sinus rhythm (SR).

**Methods:**

We examined 46 subjects (19 with AF, and 27 in SR) undergoing coronary bypass or valve surgery. Peroperative bioptic samples of the left and the right atrial tissue were examined using immunohistochemistry.

**Results:**

The number of CD3+ T-lymphocytes and CD68-KP1+ cells were elevated in the left atrial myocardium of patients with AF compared to those in SR. Immune cell infiltration of LA was related to the rhythm, but not to age, body size, LA size, mitral regurgitation grade, type of surgery, systemic markers of inflammation or presence of diabetes or hypertension. Most of CD68-KP1+ cells corresponded to dendritic cell population based on their morphology and immunoreactivity for DC-SIGN. The numbers of mast cells and CD20+ B-lymphocytes did not differ between AF and SR patients. No foci of inflammation were detected in any sample.

**Conclusions:**

An immunohistochemical analysis of samples from patients undergoing open heart surgery showed moderate and site-specific increase of inflammatory cells in the atrial myocardium of patients with AF compared to those in SR, with prevailing population of monocyte-macrophage lineage. These cells and their cytokine products may play a role in atrial remodeling and AF persistence.

## Introduction

From all cardiac arrhythmias in human, atrial fibrillation (AF) is one of the most common and important irregularities of the heart rhythm. The prevalence of AF is 1–2% in general population, but it reaches up to 10% in elderly subjects [1]. It confers approximately 5-fold higher risk of stroke; one in five of all strokes is attributed to this arrhythmia and the strokes associated with AF are more severe than strokes from other etiologies [2]. Despite its obvious clinical importance, the pathogenesis of AF remains only partially explained. The current classification of AF is arbitrary and based mainly on its duration [3]. Paroxysmal AF is defined as recurrent AF that terminates spontaneously within several days. Persistent AF is defined as AF which is sustained beyond seven days, or lasting less than seven days but necessitating pharmacologic or electrical cardioversion. Included within the category of persistent AF is “longstanding persistent AF” which is defined as continuous AF of greater than one-year duration. The term permanent AF is defined as AF in which cardioversion has either failed or not been attempted, and arrhythmia is accepted as permanent rhythm. Unfortunately, this classification does not take into account the real underlying atrial substrate and there has been a call for a mechanistic classification of AF with important therapeutic benefits [4]. The main factors predisposing to AF are aging, arterial hypertension, valvular disease, congestive heart failure and coronary artery disease [5]. Together with diabetes mellitus, lung disease, peri-and myocarditis, cardiomyopathy and thyroid disease these predisposing factors belong to well-known conditions capable of favoring this arrhythmia [5]. However, new predisposing factors are also emerging, such as obstructive sleep apnea, obesity, heavy alcohol consumption, endurance sport activities and gene mutations [5–7]. These risk factors lead to functional and morphological changes that can support maintenance of AF and are believed to be responsible for a progressive character of the arrhythmia that tends to develop over time from paroxysmal to permanent form. Three forms of atrial remodeling during a progression of AF have been described: electrical, contractile and structural [8]. Electrical remodeling is a consequence of high atrial rate and includes shortening of the refractory period of atrial cardiomyocytes and slowing the velocity of atrial conduction [9]. The structural remodeling is characterized both by changes in cardiomyocytes [10, 11] and in the endomysium [12, 13], by changes in extracellular matrix composition and atrial fibrosis [14–16]. The changes at the level of cardiomyocytes include the loss of contractile structures (“myocytolysis”), a switch to more fetal-like phenotype as manifested by altered expression of certain proteins, accumulation of glycogen and other changes at the ultrastructural level [6]. Changes in the interstitium are mainly manifested by the deposition of collagen fibers around cardiomyocytes [17]. Contractile remodeling is caused mainly by impaired calcium handling and may result in atrial mechanical dysfunction that may be transient (stunning). Impaired contractility can result not only from the changes of cell physiology, but also structural remodeling at the level of individual cardiomyocytes as well as at the level of the whole atrial tissue. The three forms of the atrial remodeling are thus interrelated [8]. Another morphological feature related to AF is the presence of inflammatory cells in the atrial myocardium [11]. Despite observed association between elevated plasma levels of inflammatory markers and AF, it is still not determined whether inflammation is a systemic or local phenomenon reflecting an active inflammatory process in the heart [18]. A role of inflammation and myocardial inflammatory infiltrate was suggested by morphological studies and by clinical studies that monitored serum levels of inflammatory cytokines in patients with AF [18, 19]. Histological findings supporting the association between inflammation and AF have been reported in several animal [20, 21] as well as human studies [11, 22–24]. Other reports have shown a correlation between the level of fibrosis and infiltration of atrial myocardium by inflammatory cells [25]. However, the above studies focused on inflammatory cells in general and were usually limited to one atrium only or to single pathological condition related to AF. It is not known whether the inflammatory cells are a marker of local reaction to tissue injury caused by factors leading to AF or whether they actively participate in the maintenance of AF due to direct cytotoxic or pro-fibrotic effects [26] or indirectly due to released cytokines that may promote arrhythmogenesis [21]. The aims of this study were the following: 1) to characterize and quantify immune cell populations in human atrial myocardium of patients undergoing open heart surgery with AF and in sinus rhythm (SR); 2) to identify regional differences in terms of immune cell populations in atrial myocardium of patients undergoing open heart surgery; 3) to correlate atrial immune cell populations with markers of systemic inflammation or AF risk factors.

## Materials and methods

### Patients

We used bioptic material from 46 patients (19 with long-term persistent AF and 27 in SR), which were hospitalized in Institute for Clinical and Experimental Medicine in Prague. We included patients who underwent coronary artery bypass surgery or valve surgery and who agreed to participate in the study (S1 Table). Patients who presented with sepsis, active endocarditis, permanent cardiostimulation, dominating atrial flutter or postincisional supraventricular tachycardia on ECG were excluded. The study conformed to the principles presented in the Declaration of Helsinki. Ethics committee of the Institute for Clinical and Experimental Medicine in Prague approved the study protocol. Only those patients who signed a written informed consent were included into the study.

### Tissue sampling, histological and immunohistochemical analysis

Tissue samples were obtained during open-heart surgery and processed as described previously [27]. Immunohistochemistry was used to visualize CD45, CD3, CD68-KP1, DC-SIGN (DC-specific ICAM-3 grabbing nonintegrin), mast cell tryptase and CD20 in these samples (S2 Table).

### Histomorphometry

To quantify CD45+cells, CD3+cells, CD68-KP1+cells and mast cells we used the program Image J 1.44p (National Institutes of Health, USA). Image sampling was performed as described in [27]. Only immunoreactive cells within the myocardium were counted manually and labeled in the image analysis program to make sure that no cell is counted twice. The frequency of cells was expressed as the number of cells per square millimeter. Those, who performed image analysis were blinded to patient characteristics.

### Statistical analysis

The values of morphometric analyses are expressed as a mean±SD. The range of values is also provided. Statistical testing of the morphometric analysis comparing values from AF and SR groups in various anatomical locations was performed using a non-parametric test—Mann–Whitney U test. Statistical significance of differences between the patient groups in various patient characteristics was tested using Student’s t-test, Fisher’s exact test, or chi-square test where appropriate. Statistical programs used for these analyses were Microsoft Office Excel, InStat and Statistica. A value of P < 0.05 was considered significant.

## Results

### Patient characteristics

S1 Table shows characteristics of the patient population in detail. The group of patients suffering from AF was 7 years older on average. Patients from AF group had higher left and right atrial volumes. Left atrial volume was on average almost doubled in subjects with AF. On the other hand, patients in SR had more often coronary artery disease. There was no difference in NYHA class, diabetes, arterial hypertension, anemia and renal insufficiency between both patient groups. E/E ‘ratio, non-invasive estimate of left atrial pressure and markers of systemic inflammation (CRP, leukocyte count) were similar between patients with AF and SR.

### Detection and quantification of CD45+ cells in the atrial myocardium

**Fig 1:**
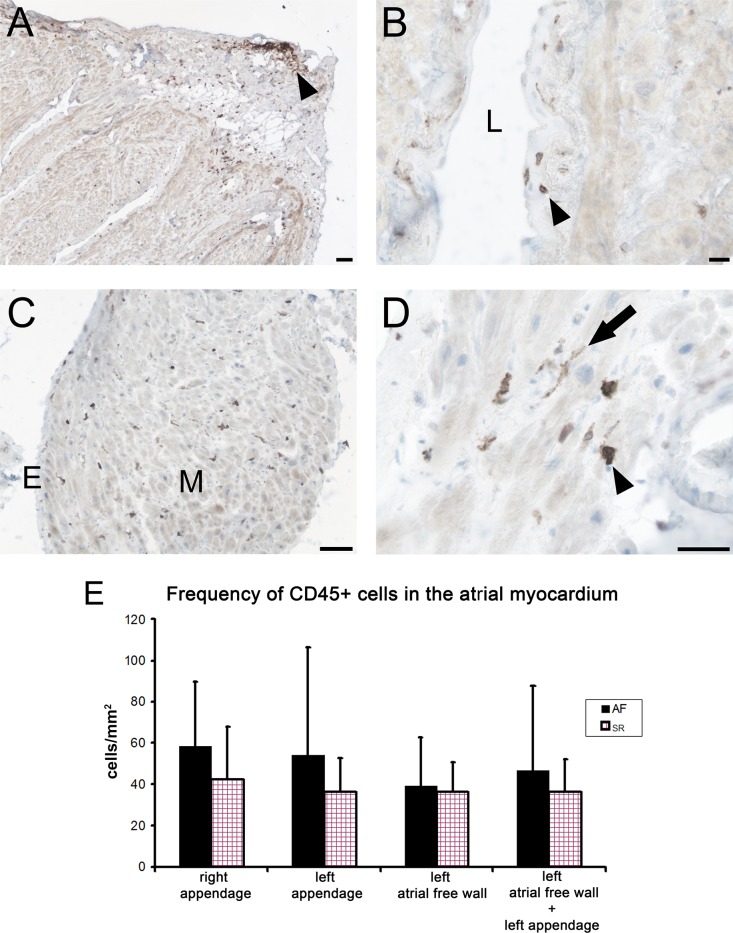
CD45-positive cells in the atrial myocardium. Sections of atrial wall from patients with sinus rhythm (A, B, D) and atrial fibrillation (C) showing a result of immunoperoxidase reaction for CD45. A) Epicardial and myocardial layer of atrial wall contains scattered as well as clustered (arrowhead) CD45-positive cells. Scale bar = 100μm. B) Endocardial layer with CD45-positive cells (arrowhead) next to the atrial lumen (L). Scale bar = 20μm. C) A section through the trabecular part of the atrial wall with CD45-positive cells in the myocardium (M) and endocardium (E). Scale bar = 50 μm. D) A detailed view on the atrial myocardium with CD45-positive cells having rather rounded (arrowhead) or elongated (arrow) cell shape. Scale bar 50 = μm. E) Frequency of CD45+ cells in the atrial myocardium of patients with atrial fibrillation (AF) and sinus rhythm (SR). An average number of CD45+ cells per square mm of cross-sectioned atrial myocardium is given +- SD. Right appendage–SR (n = 22), AF (n = 15); Left appendage—SR (n = 8), AF (n = 8); Left atrial free wall—SR (n = 4), AF (n = 8); Left atrial free wall + left appendage—SR (n = 12), AF (n = 16).

To visualize cells with a potential of an inflammatory process we used an antibody against a pan-leukocyte marker CD45. This marker is common for cells of hematopoietic origin. Immunoperoxidase reaction revealed CD45+ cells in all samples analyzed from SR and AF group. The immunoreactive cells were found in the whole thickness of the atrial wall regardless of the anatomical location (Fig 1A–1D). In the epicardium, CD45+ cells often formed larger clusters (Fig 1A). In the endocardium, these cells were scattered in the whole thickness of this layer (Fig 1B), occasionally they also adhered to the endothelium from the luminal side (not shown). The cells immunoreactive for CD45+ were found in the interstitial compartment of the myocardium both in perimysium and in endomysium. Morphologically, the CD45+ cell population appeared heterogeneous with cells having in general either rounded or elongated cell bodies (Fig 1D). The CD45+ cells located in the myocardium were quantified in tissue sections of left and right atrial appendages and left atrial free wall. When samples from SR group and AF group were compared there was a tendency for a higher frequency of CD45+ cells in AF group, however, without reaching a statistical significance (Fig 1E). In the right atrial appendage, the average number of CD45+ cells per 1mm^2^ was 42.4±25.5 (range 12.2–103.8) in the SR group and 58.4±31.3 (range 12.6–118.5) in the AF group. In the left atrial appendage, the average number of CD45+ cells per 1mm^2^ was 36.1±16.7 (range 14.4–61.7) in the SR group and 54.2±52.2 (range 17.8–186.2) in the AF group. In the left atrial free wall, the average number of CD45+ cells per 1mm^2^ was 36.2±14.2 (range 20.6–59.3) in the SR group and 39.0±23.7 (range 11.9–95.5) in the AF group. In the pooled samples from the whole left atrium, the average number of CD45+ cells per 1mm^2^ was 36.1±15.9 (range 14.3–61.7) in the SR group and 46.6±41.2 (range 11.9–186.2) in the AF group.

### CD3-positive T-lymphocytes in the atrial myocardium

**Fig 2:**
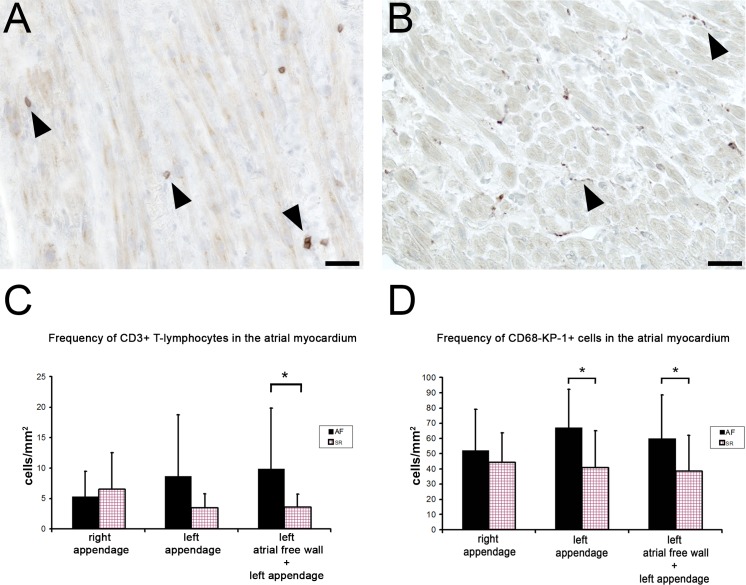
CD3-positive T-lymphocytes and CD68-KP1-positive cells in the atrial myocardium. Sections of atrial myocardium from patients with atrial fibrillation showing a result of immunoperoxidase reaction for CD3 and CD68-KP1. A) CD3-positive T-lymphocytes localized among atrial cardiomyocytes and in the interstitial spaces (arrowheads). Scale bar = 50 μm. B) CD68-KP1-positive cells are found in the interstitium and have mainly elongated morphology (arrowheads). Scale bar = 50 μm. C) Frequency of CD3-positive T-lymphocytes in the atrial myocardium of patients with atrial fibrillation (AF) and sinus rhythm (SR). An average number of CD3-positive T-lymphocytes cells per square mm of cross-sectioned atrial myocardium is given +- SD. Right appendage–SR (n = 17), AF (n = 11); Left appendage—SR (n = 8), AF (n = 10); Left atrial free wall + left appendage—SR (n = 9), AF (n = 16). *—p<0,05 D) Frequency of CD68-KP1-positive cells in the atrial myocardium of patients with atrial fibrillation (AF) and sinus rhythm (SR). An average number of CD68-KP1-positive cells per square mm of cross-sectioned atrial myocardium is given +- SD. Right appendage–SR (n = 22), AF (n = 9); Left appendage—SR (n = 11), AF (n = 9); Left atrial free wall + left appendage—SR (n = 13), AF (n = 19). *—p<0,05

With the aim to characterize the inflammatory cell population further, we performed analysis of different subpopulations of CD45+ cells. Thus, as a next step, T-lymphocytes were immunohistochemically detected in atrial samples using an antibody against a marker CD3. Like CD45+ cells, T-lymphocytes were found in all samples from both SR and AF groups and were localized in the whole atrial wall including myocardium (Fig 2), but were much less abundant than CD45+ cells. The rounded morphology of CD3+ cells, which were found scattered in endomysial and perimysial interstitial spaces corresponded to that of lymphocytes (Fig 2A). The quantification of CD3+ T-lymphocytes revealed their significantly higher frequency in left atrium of patients with AF. When samples of left atrial appendage and left atrial free wall were pooled the difference between AF and SR group was statistically significant (Fig 2C). In the right atrial appendage, the average number of CD3+ cells per 1mm^2^ was 6.5±6.0 (range 1.08–26.8) in the SR group and 5.3±4.2 (range 1.2–15.3) in the AF group. In the left atrial appendage, the average number of CD3+ cells per 1mm^2^ was 3.5±2.3 (range 1.1–8.4) in the SR group and 8.6±10.1 (range 1.7–36.2) in the AF group. In the pooled samples from the whole left atrium, the average number of CD3+ cells per 1mm^2^ was 3.5±2.2 (range 1.1–8.4) in the SR group and 9.8±10.0 (range 1.7–36.2) in the AF group.

### CD68-KP1-positive cells in the atrial myocardium

Inflammatory cells including monocyte/macrophages and dendritic cells can be detected using a marker CD68-KP1. Cells immunoreactive for CD68-KP1 were very frequent in all atrial tissue samples from both AF and SR group. They were regularly detected in the myocardium and had predominantly an elongated cell shape (Fig 2B). The quantitative analysis revealed that CD68-KP1+ cell population was the most abundant inflammatory cell population and they were more frequent in samples from patients with AF (Fig 2D). There was significantly higher number of CD68-KP1+ cells in the left appendage and in pooled samples of left appendage and left atrial wall in patients with AF compared to patients with SR (Fig 2D). In the right atrial appendage, the average number of CD68-KP1+ cells per 1mm^2^ was 44.3±19.3 (range 7.7–76.7) in the SR group and 52.0±27.0 (range 14.0–109.0) in the AF group. In the left atrial appendage, the average number of CD68-KP1+ cells per 1mm^2^ was 40.8±24.4 (range 9.9–96.3) in the SR group and 67.2±25.0 (r[7]ange 41.6–122.4) in the AF group. In the pooled samples from the whole left atrium, the average number of CD68-KP1+ cells per 1mm^2^ was 38.5±23.7 (range 9.9–96.3) in the SR group and 60.2±28.4 (range 18.0–122.4) in the AF group.

### DC-SIGN-positive dendritic cells, mast cells and B-lymphocytes in the atrial myocardium

**Fig 3:**
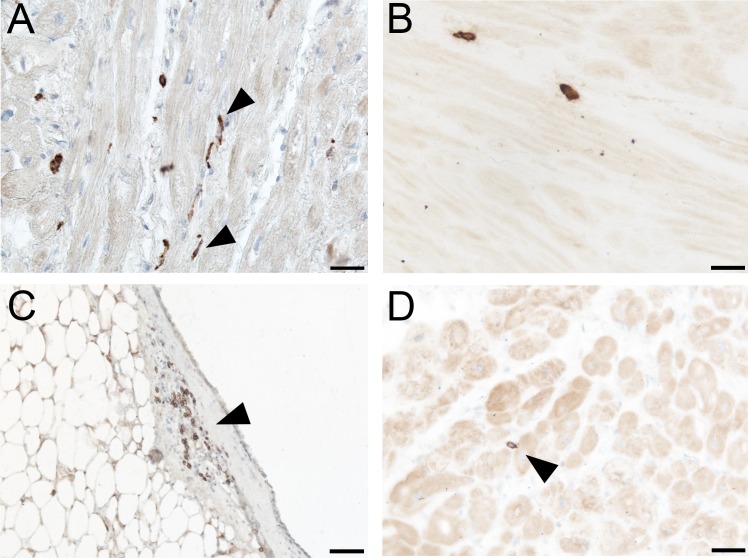
DC-SIGN-positive cells, mast cells and B-lymphocytes in the atrial wall. A) A section of atrial myocardium from a patient with atrial fibrillation showing a result of immunoperoxidase reaction for DC-SIGN. DC-SIGN—positive cells have mainly elongated morphology (arrowheads). Scale bar = 50 μm. B) A section of atrial myocardium from a patient with sinus rhythm. Mast cells detected using anti-mast cell tryptase antibody are scattered in the atrial myocardium. Scale bar = 50 μm. (C-D) Sections of atrial wall from a patient with sinus rhythm. C) A cluster of CD20-positive B-lymphocytes in the epicardial layer (arrowhead). Scale bar = 100 μm. D) A detail of atrial myocardium with an isolated CD20-positive B-lymphocyte. Scale bar = 50 μm.

Dendritic cells are among cells immunoreactive for CD68-KP1+. To confirm their presence in the atrial myocardium of patients undergoing open heart surgery we performed an immunohistochemical detection of marker DC-SIGN, which labels immature dendritic cells. DC-SIGN+ cells were found frequently in atrial myocardial samples from both AF and SR groups. They had the same morphology as the cells previously immunoreactive for CD68-KP1 antigen (Fig 3A). Mast cells were detected using an antibody against mast cell tryptase.

Mast cells were quite rare in the atrial myocardium and they were found scattered as individual cells in the endomysial compartment or in small groups in the perimysial spaces surrounding bigger vessels (Fig 3B). There was a similar frequency of mast cells in the atrial myocardium of patients in AF and SR group (not shown). B-lymphocytes were detected using an antibody against CD20 marker in the atrial myocardium of patients in AF and SR group. CD20+ cells were occasionally found as small clusters in the epicardial layer (Fig 3C). In the myocardium, these cells were very rare (Fig 3D).

### Determinants of inflammatory cell populations other than cardiac rhythm

In the pooled cohort, variations of immune cell population were related to prevailing rhythm (particularly for CD3 and CD68-KP1 markers). However, they did not significantly correlate with mitral regurgitation grade, left atrial volume, E/E´ ratio, age, presence of diabetes, hypertension or coronary artery disease. Inflammatory infiltrate was related to the rhythm itself, rather than to risk factors known to be associated with AF. We also did not observe significant correlation between atrial inflammatory infiltration by CD45+, CD3+ or CD68-KP1+ and CRP or blood leukocyte count.

## Discussion

The results of this study can be summarized as follows: 1) The present study shows that CD45+ cells are a common finding in atrial myocardium of patients undergoing open heart surgery regardless of the actual heart rhythm; 2) An analysis of CD45+ cell count was performed in different anatomical locations–left atrial appendage, left atrial free wall and right atrial appendage; 3) There was a tendency for a higher CD45+ cell count in samples from patients with AF and this difference was even more prominent when focusing on some specific subpopulations of inflammatory cells in the atrial myocardium; 4) Both in case of CD3+ T-lymphocytes and CD68-KP1+ cells there was a higher cell count in the samples from patients with AF, but only in the left atrium.

Several studies have been published previously describing an elevated count of inflammatory cells in the atria of patients suffering from AF [11, 15, 22–25, 28, 29]. In contrast to our study, some papers evaluated samples only from either left or right atrium [22, 24, 28, 29] or the analysis was performed on necroptic specimens [25]. The results of our study are in general agreement with findings of the above studies performed on different patient populations. Importantly, it shows that there is significantly larger immune cell count only in the left atrium of patients suffering from AF. The reason for the differential response of atria in terms of inflammatory cell infiltration might be explained by a differences in density of capillaries serving as an entrance port for immune cells to invade atrial interstitium. In our recent study performed on the same patient groups, we reported a higher microvascular density in the left atrium compared to the right atrium [27]. It was shown repeatedly that the most pronounced morphological changes attributable to AF could be detected in the left posterior atrial wall [17]. Since, in the present study, most samples of the left atrium originated from the left appendage and a smaller number from left atrial free wall, we could not evaluate them separately, however the statistical significance of differences between CD3+ and CD68-KP1+ always increased when the samples from both locations were pooled together.

In addition to above mentioned immune cell populations, we also evaluated the amount of mast cells and B-lymphocytes. One experimental study suggested the crucial role of mast cells for development of AF [21]. However, in our study we found only a small number of mast cells in the atrial myocardium and there was no difference between AF and SR groups of patients.

Our data show rather moderate increase of immune cells in left atrial myocardium of patients with AF compared to SR group of patients undergoing open heart surgery. In case of CD3+ T-lymphocytes, we found diffuse infiltration mostly by isolated cells. Interestingly, in contrast to an initial report [11], no inflammatory infiltrates were found in more recent study analyzing atrial myocardium from patients with lone atrial fibrillation [7]. In the present study, we were not able to detect active (acute) myocarditis according to current criteria [30, 31]. It seems likely that an elevated number of CD3+ and CD68-KP1+ cells in the left atrial myocardium of patients with AF reflects certain level of inflammatory activation of myocardial tissue due to the mechanical stress accompanying the atrial dilation [32, 33]. Expression of inflammatory cytokines is another parameter of local inflammatory state. One study reported that there was an increased production of TNF-alpha and IL-6 in the right atria of AF patients with valvular disease compared to control group of SR patients with valvular disease [34]. A more recent study demonstrated a higher expression of an inflammatory cell adhesion molecule VCAM1 in myocardial capillaries of left atrial appendages of patients with AF compared with autoptic SR controls [29]. Interestingly, while the former study also reported a higher collagen volume fraction in patients with AF [34], no difference between AF patients and controls was observed in the latter [29]. Fibrosis is considered by many authors as a hallmark of structural remodeling in AF [13–16] and can also cause remodeling of infiltrating subepicardial fatty infiltrates [35]. However, there are also reports, where no statistical difference in collagen volume fraction or fibrosis was found between AR and SR group of patient most likely due to a variable influence of accompanying structural heart diseases [27, 29, 32, 36, 37]. The largest immune cell population was formed by CD68-KP1+ cells of monocyte/macrophage lineage corresponding largely to dendritic cells, which were immunoreactive to DC-SIGN. The role of these cells that are also found in the normal myocardium [38] is still a matter of ongoing research and these cells can have both beneficial as well as detrimental effects on the heart, based on various pathophysiological circumstances [39, 40]. In a recent study it was demonstrated that atrial myocarditis coincides with certain types of ventricular myocarditis [41], but since we have no reports of ventricular myocarditis in our patients we are unable to relate an increased number of CD3+ and CD68-KP1+ cells in the left atria of AF group to an inflammatory process in other parts of the myocardium. Our goal was to quantify inflammatory cells in the atrial myocardia. Although we observed inflammatory cells regularly in the endocardium and epicardium regardless of the heart rhythm we did not perform a quantitative study in these layers of the heart wall and thus future studies may demonstrate whether there is a correlation between the number of these cells across the heart. There are several limitations resulting from the fact that we relied on material from bioptic samples. In addition, the small size of our patient groups might affect our quantitative results. It was possible to safely harvest only small bioptic samples and these could be obtained from only certain regions within the atria. Only patients suffering from various structural heart diseases were compared.

## Conclusions

Immunohistochemical analysis of tissue samples from patients undergoing open heart surgery showed moderate and site-specific increase of inflammatory cells in the atrial myocardium of patients with AF compared to those in SR. The most represented immune cell population was CD3+ T-lymphocytes and CD68-KP1+ monocyte/macrophage subpopulation corresponding mostly to dendritic cells. Mast cells and B-lymphocytes were less frequent. Only CD3+ and CD68-KP1+ cells were elevated in the left atrium of patients with AF, while no inflammatory foci were detected in the atrial myocardium. This local elevation of some CD45+ cell populations might reflect the progression of AF. More research has to be done to elucidate significance of general pro-inflammatory state reported in patients with AF.

## Supporting information

S1 Table Characteristics of patients with AF and patients in SR. (DOCX)

S2 Table Characterization of antibodies used in the study. (DOC)
