# Supplementary material for: Analysis of immune cell populations in atrial myocardium of patients with atrial fibrillation or sinus rhythm
Source: PLoS One. 2017 Feb 22;12(2):e0172691. doi: 10.1371/journal.pone.0172691 (PMC5321459; doi:10.1371/journal.pone.0172691)
Supplement: S1 Table — (DOCX) [file pone.0172691.s001.docx]

**S1 Table. Characteristics of patients with AF and patients in SR**

| **Characteristic** | **Total** | **AF** | **SR** | **P value** |
| --- | --- | --- | --- | --- |
|  | (n=46) | (n=19) | (n=27) |  |
| Age (years) | 65±9 | 69±7 | 62±9 | 0.0092 |
| Sex (m/f) | 25/21 | 13/6 | 12/15 | n.s. |
| Height (cm) | 169±10 | 170±10 | 169±10 | 0.7280 |
| Weight (kg) | 84±19 | 84±21 | 83±15 | 0.5622 |
| Atrial fibrillation duration (years) | --- | 8.3±11.1 | --- | --- |
| Obesity | 19(41.3%) | 7(36.8%) | 12(44.4%) | n.s. |
| Diabetes mellitus | 10(21.7%) | 4(21.1%) | 6(22.2%) | n.s. |
| Arterial hypertension | 37(80.4%) | 16(84.2%) | 21(77.8%) | n.s. |
| Inflammation (incl. pericarditis, sepsis, systemic inflammatory disease) | 0 (0%) | 0 (0%) | 0 (0%) | n.s. |
| Prior pericardiac effusion | 9 (19.6%) | 5 (26.3%) | 4 (14.8%) | n.s. |
| Serum Creatinine (µmol/l) | 88.10±23.06 | 93.86±27.82 | 84.26±18.87 | 0.1742 |
| C-reactive protein, g/l | 4.2 ± 5.8 | 3.1 ± 4.0 | 5.0 ± 6.8 | n.s. |
| Leukocyte count, *10^6^/ml | 7.2 ± 1.7 | 7.0 ± 2.1 | 7.3 ± 1.5 | n.s. |
| Hemoglobin, g/l | 137 ± 16 | 134 ± 16 | 138 ± 16 | n.s. |
| Hemoglobin A1C, % | 4.6 ± 1.5 | 4.8 ± 1.7 | 4.5 ± 1.4 | n.s. |
| CHA2DS2-VASc score | 3.35±1.86 | 3.30±1.30 | 3.42±1.50 | 0.7649 |
| NYHA class |  |  |  |  |
| <0-1.5) | 3 (8.1%) | 1 (6.3%) | 2 (9.5%) | n.s. |
| <1.5-2.5) | 16 (43.2%) | 7 (43.7%) | 9 (42.9%) | n.s. |
| <2.5-3.5) | 18 (48.7%) | 8 (50%) | 10 (47.6%) | n.s. |
| <3.5-4> | 0 (0%) | 0 (0%) | 0 (0%) | n.s. |
| total | 37 (100%) | 16 (100%) | 21 (100%) |  |
| Coronary artery disease | 32(69.6%) | 8(42.1%) | 24(88.9%) | 0.001 |
| Mitral valve stenosis (data available in 41/46 pts.) |  |  |  |  |
| none or mild (grade 0-2/4) | 40 (97.6%) | 15 (93.8%) | 25 (100%) | n.s. |
| moderate (grade 3/4) | 1 (2.4%) | 1 (6.2%) | 0 (0%) | n.s. |
| severe (grade 4/4) | 0 (0%) | 0 (0%) | 0 (0%) | n.s. |
| total | 41 (100%) | 16 (100%) | 25 (100%) |  |
| Mitral valve regurgitation (data available in 41/46 pts.) |  |  |  |  |
| none or mild (grade 0-2/4) | 30 (73.2%) | 7 (43.8%) | 23 (92.0%) | 0.003 |
| moderate (grade 3/4) | 3 (7.3%) | 2 (12.4%) | 1 (4.0%) | 0.003 |
| severe (grade 4/4) | 8 (19.5%) | 7 (43.8%) | 1 (4.0%) | 0.003 |
| total | 41 (100%) | 16 (100%) | 25 (100%) |  |
| Aortic valve stenosis (data available in 42/46 pts.) |  |  |  |  |
| none or mild (grade 0-2/4) | 32 (76.2) | 14 (82.3%) | 18 (72.0%) | n.s. |
| moderate (grade 3/4) | 2 (4.8%) | 1 (5.9%) | 1 (4.0%) | n.s. |
| severe (grade 4/4) | 8 (19.0%) | 2 (11.8%) | 6 (24.0%) | n.s. |
| total | 42 (100%) | 17 (100%) | 25 (100%) |  |
| Aortic valve regurgitation (data available in 40/46 pts.) |  |  |  |  |
| none or mild (grade 0-2/4) | 33 (82.5%) | 10 (66.7%) | 23 (92.0%) | 0.0245 |
| moderate (grade 3/4) | 4 (10.0%) | 4 (26.7%) | 0 (0%) | 0.0245 |
| severe (grade 4/4) | 3 (7.5%) | 1 (6.6%) | 2 (8.0%) | 0.0245 |
| total | 40 (100%) | 15 (100%) | 25 (100%) |  |
| Tricuspid valve regurgitation (data available in 40/46 pts.) |  |  |  |  |
| none or mild (grade 0-2/4) | 33 (82.5%) | 8 (53.3%) | 25 (100%) | 0.00085 |
| moderate (grade 3/4) | 4 (10%) | 4 (26.7%) | 0 (0%) | 0.00085 |
| severe (grade 4/4) | 3 (7.5%) | 3 (20.0%) | 0 (0%) | 0.00085 |
| total | 40 (100%) | 15 (100%) | 25 (100%) |  |
| LV diameter | 54.0±7.0 | 55.44±8.0 | 53.0±6.1 | 0.2548 |
| LV EF (%) | 51.6±10.4 | 49.5±11.7 | 53.1±9.3 | 0.3000 |
| LA volume | 93.4±51.4 | 121.3±62.5 | 73.0±28.5 | 0.0003 |
| RA volume | 71.0±33.0 | 84.2±39.5 | 61.3±23.8 | 0.0202 |
| E/E’ ratio | 13± 7.5 | 14 ± 8 | 13 ± 7 | n.s. |
| **Type of surgery** |  |  |  |  |
| CABG | 32(69.6%) | 8(42.1%) | 24(88.9%) | 0.001 |
| Mitral valve repair or replacement | 12(26.1%) | 8(42.1%) | 4(14.8%) | 0.05 |
| Aortic valve replacement | 17(37.0%) | 8(42.1%) | 9(42.9%) | n.s. |

LV, left ventricle; EF, ejection fraction; LA, left atrium; RA, right atrium; CABG, coronary artery bypass surgery; CHA2DS2-VASc (The Congestive heart failure, Hypertension, Age ˃75, Diabetes mellitus, Stroke, Vascular disease, Age 65-74 years, Sex category) stroke risk assessment in Atrial Fibrillation; n.s.: not significant. For frequency distributions p-value is based on chi-square test of all categories. Values are expressed as average ±SD.
