# Supplementary material for: Analysis of immune cell populations in atrial myocardium of patients with atrial fibrillation or sinus rhythm
Source: PLoS One. 2017 Feb 22;12(2):e0172691. doi: 10.1371/journal.pone.0172691 (PMC5321459; doi:10.1371/journal.pone.0172691)
Supplement: S2 Table — (DOC) [file pone.0172691.s002.doc]

**S2 Table.** **Characterization of antibodies used in the study**

**Antibody**

**Abbreviation**

**Catalog ID/Lot**

**ID**

**Dilution**

**Retrieval**

**Clone/Isotype**

**Produced by**

Monoclonal Mouse Anti-Human

CD45 Leucocyte Common Antigen

CD45

M0701

1:100

Tris buffer + EDTA

1A4 IgG2a

DakoCytomation,

Glostrup, Denmark

Polyclonal Rabbit Anti – Human CD3

CD3

A0452

1:8000

Tris buffer + EDTA

DakoCytomation,

Glostrup, Denmark

Monoclonal Mouse Anti-Human

CD68

CD68

M0814

1:200

Tris buffer + EDTA

DakoCytomation,

Glostrup, Denmark

Monoclonal Mouse Anti-Human

Mast cell Tryptase

MCT

M7052

1:6000

Tris buffer + EDTA

D33 IgG1

DakoCytomation,

Glostrup, Denmark

DC-SIGN

(Rabbit Anti-Human polyclonal)

CD20

M0755

1:400

Tris buffer + EDTA

LifeSpan BioSciences,

USA

DC-SIGN

LS-B479

1:6000

Tris buffer + EDTA

Monoclonal Mouse Anti-Human

CD20

DakoCytomation,

Glostrup, Denmark

L26 IgG2a, kappa

KP1 IgG1
